# Parental presence and intranasal dexmedetomidine for the prevention of anxiety during anesthesia induction in children undergoing tonsillectomy and/or adenoidectomy surgery: A randomized controlled trial

**DOI:** 10.3389/fphar.2022.1015357

**Published:** 2022-12-19

**Authors:** Jing Yao, Hesong Gong, Xiaochun Zhao, Qinxue Peng, Hongjuan Zhao, Shuangshuang Yu

**Affiliations:** ^1^ Department of Anesthesiology, Cancer Hospital of China Medical University, Liaoning Cancer Hospital & Institute, Shenyang, China; ^2^ Department of Anesthesiology, Shengjing Hospital of China Medical University, Shenyang, China; ^3^ Department of Anesthesiology, School and Hospital of Stomatology, China Medical University, Shenyang, China; ^4^ Department of Anesthesiology, Shenzhen Hospital of Southern Medical University, Shenzhen, China; ^5^ Department of Anesthesiology, The Third Affiliated Hospital of Shenyang Medical College, Shenyang, China; ^6^ Department of Anesthesiology, The Fifth Affiliated Hospital of Sun Yat-sen University, Zhuhai, China

**Keywords:** parental presence at induction of anesthesia, dexmedetomidine, preoperative anxiety, emergence delirium, perioperative

## Abstract

**Background:** During the perioperative period of pediatric surgery, it is extremely stressful for children and parents to enter the operating room and receive the anesthesia induction. This study was designed to evaluate the perioperative outcomes with parental presence at induction of anesthesia (PPIA), intranasal dexmedetomidine, and combined use of PPIA and intranasal dexmedetomidine.

**Methods:** In this prospective study, 124 children were randomly divided into four groups: control (no parental presence or intranasal dexmedetomidine), PPIA (parental presence), DEX (intranasal dexmedetomidine (1.0 μg/kg)), and PPIA + DEX (parental presence and intranasal dexmedetomidine (1.0 μg/kg)). The anxiety of children was mainly evaluated by the modified Yale Preoperative Anxiety Scale-Short Form (mYPAS-SF). Secondary evaluation methods were, for example, the Induction Compliance Checklist (ICC), the Pediatric Anesthesia Emergence Delirium Scale (PAED), the COMFORT Behavior Scale (COMFORT-B Scale), the State-Trait Anxiety Inventory (STAI), and the Visual Analog Scale (VAS).

**Results:** Children in the PPIA + DEX group exhibited significantly lower mYPAS-SF and ICC scores compared with all three other groups (*p* < 0.001), and children in that group exhibited significantly lower mYPAS-SF and ICC scores compared with the PPIA and DEX groups (*p* < 0.05). The children’s PAED scores in the PPIA, DEX, and PPIA + DEX groups were significantly lower than the control group (*p* < 0.001).The STAI-S scores of the PPIA, DEX, and PPIA + DEX groups were significantly lower than the score of the control group (*p* < 0.001). The VAS scores of the PPIA, DEX, and PPIA + DEX groups were significantly higher than that of the control group (*p* < 0.001), while the score of the PPIA + DEX group was significantly higher than those of the PPIA and DEX groups (*p* < 0.05).

**Conclusion:** The combined use of PPIA and intranasal dexmedetomidine is more effective than PPIA or intranasal dexmedetomidine for alleviating the preoperative anxiety of children, improving children’s induction compliance and parental satisfaction.

## 1 Introduction

The perioperative period of pediatric surgery is extremely stressful for children and their families when entering the operating room (OR) for anesthesia and surgery. Implementing a steady induction of anesthesia is challenging for anesthesiologists. Stressful induction of anesthesia causes anxiety. During anesthesia induction, more than 40% of children express pain and anxiety, and about 17% try to escape, verbally protest, cry, scream, or are scared or fearful; more than 30% refuse the induction of anesthesia by the anesthesiologist (Chorney and Kain, 2009). The adverse psychological reactions and behaviors caused by preoperative anxiety can cause both short- and long-term traumatic consequences for children. In the short term, they may experience airway spasm, respiratory circulatory dysfunction, and emergence delirium during the induction period; these may affect the safety of anesthesia. Long-term injuries are psychological and traumatic, including postoperative behavioral disorders such as nightmares, eating problems, fear of doctors, separation anxiety, loss of temper, and bedwetting (Kain et al., 1996; Fortier et al., 2010). Many parents also state that they are very anxious before their children’s surgery (Kain et al., 1996).

It is therefore imperative to alleviate the anxiety levels of children and their parents during the induction of anesthesia. Anesthesiologists have many ways to do this, such as preoperative sedatives, parental presence at induction of anesthesia (PPIA), clown doctors, transport in a toy car, tablet games, cartoon videos, and comical information leaflets (Arai et al., 2007; Lee et al., 2012; Kim et al., 2015; Kassai et al., 2016; Marechal et al., 2017; Liu et al., 2018; Lopes-Júnior et al., 2020). Some interventions are used frequently while others are rarely used because they consume too much manpower and resources or lead to additional costs. PPIA and preoperative sedatives are simple and effective, and many doctors use sedative drugs and PPIA either alternately or simultaneously to treat children’s anxiety induced by general anesthesia.

Some studies have shown that both PPIA and preoperative sedatives are useful and that PPIA makes sedative drugs more effective (Arai et al., 2007). The purpose of this study is to determine whether PPIA or intranasal dexmedetomidine are effective, and whether they are more effective alone or in combination in reducing anxiety in children and parents, lowering the degree of emergence delirium, and improving parental satisfaction and children’s compliance.

## 2 Materials and methods

This study was approved by the Chinese Clinical Trial Registry (registration number ChiCTR1800014751) and the Ethics Committee of the Shengjing Hospital of China Medical University (2017PS07K). Written informed consent was obtained from all participants the day before surgery.

### 2.1 Study population

All parents provided written informed consent. A total of 124 children aged 2–6 years—American Society of Anesthesiologists (ASA) physical status I—about to undergo ENT surgery (tonsillectomy and/or adenoidectomy) with general anesthesia were enrolled, regardless of gender. Exclusion criteria were chronic illness, developmental delay, severe functional organ (heart, liver, lung, and kidney) disease, neuropsychiatric disease, cancer, previous anesthetic and surgery experience, emergency surgery, and not volunteering or refusing to cooperate.

### 2.2 Randomization and preoperative management

The children were randomly divided into a control group, PPIA group, DEX group, and PPIA + DEX group through a computer-generated random list sealed-envelope technique by a research member (HS.G). A blinded observer (QX.P.) took charge of T0, T1, T2 evaluation, while another (HJ.Z) took charge of T3, T4 evaluation. HR and oxygen saturation (SpO2) were monitored by portable pulse oximetry during patient transfer from the holding to the operating rooms.

In the control group, the children went into the holding room without a parent and were transferred to the OR on a gurney. They were induced by inhalation anesthesia with 7% sevoflurane in 50% oxygen in the OR without parental presence.

In the PPIA group, a parent went into the holding room, and the children were induced by inhalation anesthesia with 7% sevoflurane in 50% oxygen in a parent’s arms in the holding room. They were then transferred to the OR on a gurney with 7% sevoflurane in 50% oxygen continuous inhalation after losing consciousness. The parent stayed in the waiting area.

In the DEX group, a parent went into the holding room and children were given intranasal dexmedetomidine (1.0 μg/kg) 30 min in the holding room before induction. After 30 min, they were separated from their parents and transferred to the OR on a gurney for inhalation anesthesia with 7% sevoflurane in 50% oxygen. The parent stayed in waiting area.

In the PPIA + DEX group, a parent went into the holding room and the children were given intranasal dexmedetomidine (1.0 μg/kg) 30 min in the holding room before induction. After 30 min, the children were induced by inhalation anesthesia with 7% sevoflurane in 50% oxygen in a parent’s arms in the holding room and transferred to the OR on a gurney with 7% sevoflurane in 50% oxygen continuous inhalation after losing consciousness. The parent stayed in waiting area.

### 2.3 Anesthesia induction and tracheal intubation

When the children lost consciousness, they continued to receive inhalation anesthesia with sevoflurane (1.5–2.5%) in 50% oxygen in the OR. After achieving a minimum alveolar concentration (MAC) value of 1.0, the children received sufentanil 0.3 μg/kg, etomidate 0.2 mg/kg, cisatracurium 0.2 mg/kg, dexamethasone 0.1 mg/kg, and ketorolac 0.5 mg/kg. During the operation, sevoflurane, oxygen, and nitrous oxide were used to maintain anesthesia and the MAC value was maintained between 1.1 and 1.3, with the PetCO_2_ between 35 and 45 mmHg. At the end of the surgery, sevoflurane was discontinued, the children’s secretions were aspirated, and the tracheal tube was extubated after the children awoke, with adequate spontaneous ventilation; the children were then sent to the post-anesthesia care unit (PACU). There, the children were continuously observed, before being transferred to the ward from the PACU when an Aldrete wake-up score greater than or equal to 9 was achieved (Alderte, 1995).

### 2.4 Outcome measures

**FIGURE 1:**
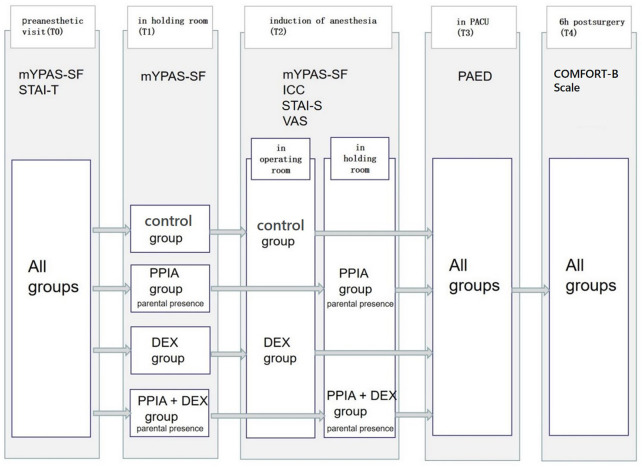
Study timeline. mYPAS-SF (modified Yale Preoperative Anxiety Scale-Short Form); ICC (Induction Compliance Checklist); STAI-T (State-Trait Anxiety Inventory-Trait); STAI-S (State-Trait Anxiety Inventory-State); PAED (Pediatric Anesthesia Emergence Delirium Scale); COMFORT-B Scale (The COMFORT Behavior Scale); VAS (Visual Analog Scale).

Five evaluation time points were set (Figure 1): the preanesthetic visit (the day before surgery) (T0); in the holding room (T1); induction of anesthesia (T2); in the PACU (T3); 6 h post-surgery (T4). At T0, T1, and T2, children’s anxiety was evaluated by the modified Yale Preoperative Anxiety Scale-Short Form (mYPAS-SF). The Induction Compliance Checklist (ICC) was used to evaluate children’s compliance at T2. The State-Trait Anxiety Inventory–Trait Anxiety (STAI-T) was completed by the parents regarding their own anxiety level at T0. At T2, after the children were transferred to the OR, the parents staying in the waiting area completed the State-Trait Anxiety Inventory–State Anxiety (STAI-S). The Visual Analog Scale (VAS) evaluated the parents’ satisfaction with the induction of the anesthesia process. At T3, the Pediatric Anesthesia Emergence Delirium Scale (PAED) was used to assess the postoperative emergence delirium of the children at T3. The COMFORT Behavior Scale (COMFORT-B Scale) was used to assess the comfort level of the children at T4.

The occurrence of bradycardia, hypotension, hypoxemia, mild adverse reactions (breath-holding, coughing, vomiting, increased respiratory secretions) and severe adverse reactions (laryngeal spasm, asthma, arrhythmia, shock, allergy, malignant fever, epilepsy, etc.) during the induction of anesthesia were recorded.

#### 2.4.1 Primary outcome measurement

The mYPAS-SF contains 18 items in four categories (children’s activity, emotional expressivity, state of arousal, and vocalization). The score ranged from 23 to 100 points. The higher the score, the more obvious the anxiety. The scale was used to assess the anxiety of the children and demonstrated strong internal reliability—the Cronbach’s *α* for the item set (four items) during each time point was at least 0.92 (Jenkins et al., 2014).

#### 2.4.2 Secondary outcome measurement

The ICC is an observational scale that includes 11 descriptions of negative behaviors related to anesthesia induction, with one point for each item. A score of 0 suggests best compliance (no negative behavior), and higher scores indicate less compliance. The highest score is ten points. The scale has high internal (0.998) and external consistency (0.987), and has excellent reliability (*k* = 0.995–0.998) and validity (*r* = 0.978) (Varughese et al., 2008).

The PAED was used to measure the children’s emergence delirium in the PACU. It assesses five behavioral items that are rated on a four-point Likert-type scale (0–20 points) to measure postanesthetic delirium during the PACU. The higher the score, the more obvious the emergence delirium (Sikich and Lerman, 2004).

The COMFORT-B Scale is an assessment tool used by Dr Van Dijk of the Netherlands to measure pain, sedation, and depression in children after modifying the original COMFORT scale. The scale consists of six behavioral indicators: alertness, calmness or emergence delirium, crying, limb movement, muscle tone, and facial tension. Each item is worth five points, with a total score of 6–30 points. The higher the score, the more comfortable the children are (van Dijk et al., 2005).

The STAI is a scale used to assess adult anxiety status, including the STAI-T and STAI-S subscales, which are used to assess trait anxiety (relatively stable anxiety tendency) and state anxiety (short anxiety state). Each subscale has 20 questions, with a score range of 20–80 points. The STAI is widely used and is a valid and reliable instrument (Cao and Liu, 2015).

In the VAS, 0 is unsatisfied and 10 is very satisfied. The parents scored themselves based on their satisfaction level. The VAS for satisfaction is a simple and valid instrument for quantifying a patient’s satisfaction after a treatment (Brokelman et al., 2012).

### 2.5 Sample size determination and statistical analysis

This study aimed to compare differences in the degree of anxiety in children. The primary outcome of this study was children’s anxiety at anesthesia induction as measured by the mYPAS-SF. A prior study of 40 children in the OR had mean (SD) mYPAS-SF scores of 88.6 (15.34), 51.4 (27.91), 52.4 (20.49), and 27 (12.65) in the control, PPIA, DEX, and PPIA + DEX groups, respectively. It was ascertained that 28 patients were required in each group to show a difference with a significance level of 0.05 (*α* = 0.05) and a power of 90% (*β* = 0.10). Sample size was calculated by one-way analysis of variance (ANOVA); a total of 112 subjects were needed to complete this study. Given the 10% rejection rate, 31 cases were enrolled for each group.

Statistical analysis was performed using SPSS 26.0 (IBM Corp, Armonk, NY). The characteristics of the children were analyzed by ANOVA and chi-squared test. Measurement data following normal distribution were expressed as mean ± SD (standard deviation). The comparison between groups was analyzed by ANOVA, and the difference between groups was compared by the LSD test. The measurement data of the skewed distribution was expressed as the median IQR (range), and comparison between groups was performed using the non-parametric Kruskal–Wallis H test. Scores such as mYPAS-SF and ICC were also compared using this test, and the difference between groups was compared using the Bonferroni test. The within-group change in mYPAS-SF scores over time was compared using the Friedman test; *p* < 0.05 was considered statistically significant.

## 3 Results

**FIGURE 2:**
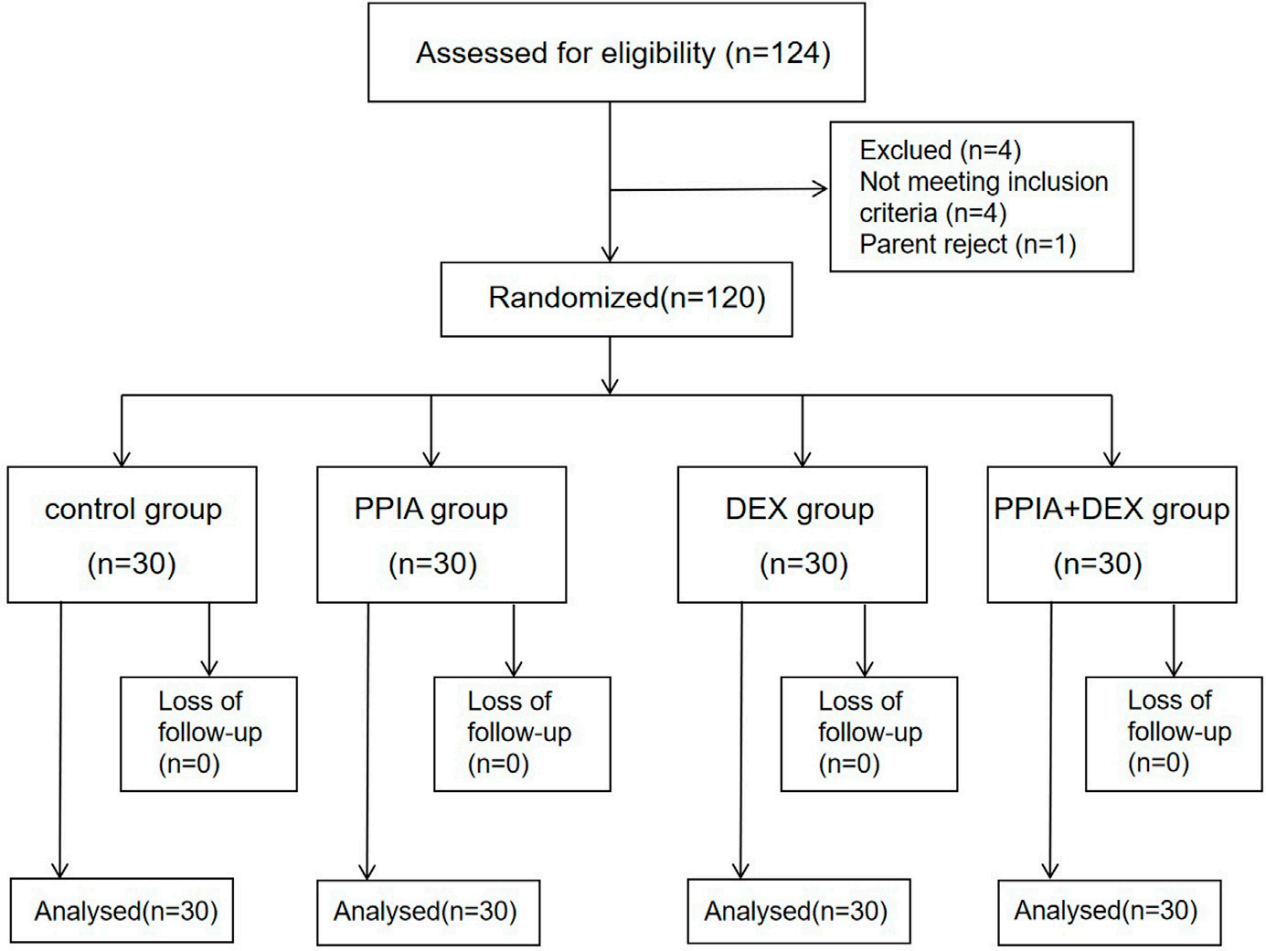
Study flow diagram. PPIA: parental presence during induction of anesthesia. DEX: intranasal dexmedetomidine.

From February to May 2018, 124 children were enrolled in the study. Three children were excluded per the study guidelines and one was excluded as the family refused to participate. Thus, 120 participants completed the study, and they were included in the data analysis (Figure 2). There were no significant differences in age, weight, height, BMI, sex, type of surgery, operation time, and recovery time among the four groups (*p* > 0.05) (Table 1).

**TABLE 1:** Characteristics of children.

Group	Control (*n* = 30)	PPIA (*n* = 30)	DEX (*n* = 30)	PPIA + DEX (*n* = 30)	Effect size F or χ2	^a^ *p*-value
Age (y)	4.3 ± 1.1	4.6 ± 1.2	4.4 ± 1.2	4.6 ± 1.4	0.579	0.630
Body weight (kg)	19.9 ± 4.5	20.9 ± 4.5	18.4 ± 4.9	19.7 ± 5.3	1.365	0.257
Body height (cm)	108.2 ± 10.7	111.4 ± 11.1	108.2 ± 9.6	109.6 ± 10.4	0.667	0.656
BMI	14.1 ± 3.4	14.3 ± 3.2	15.7 ± 3.1	14.1 ± 3.1	2.984	0.187
Gender (M/F)	20/10	18/12	18/12	16/14	0.137	0.938
Type of surgery (tonsillectomy/tonsillectomy and adenoidectomy)	19/11	15/15	16/14	19/11	0.575	0.637
Operation time	34.87 ± 1.03	36.40 ± 7.20	36.00 ± 7.29	37.77 ± 8.89	0.796	0.499
Recovery time	37.23 ± 7.71	40.20 ± 7.28	40.37 ± 7.61	42.23 ± 6.78	1.342	0.264

Data are mean ± SD.

^a^ *p*-value, result by one-way analysis of variance (ANOVA) and chi-squared test.

There were no significant differences in age, weight, height, BMI, sex, type of surgery, operation time, or recovery time among the four groups.

(*p* > 0.05).

### 3.1 Primary outcome

**FIGURE 3:**
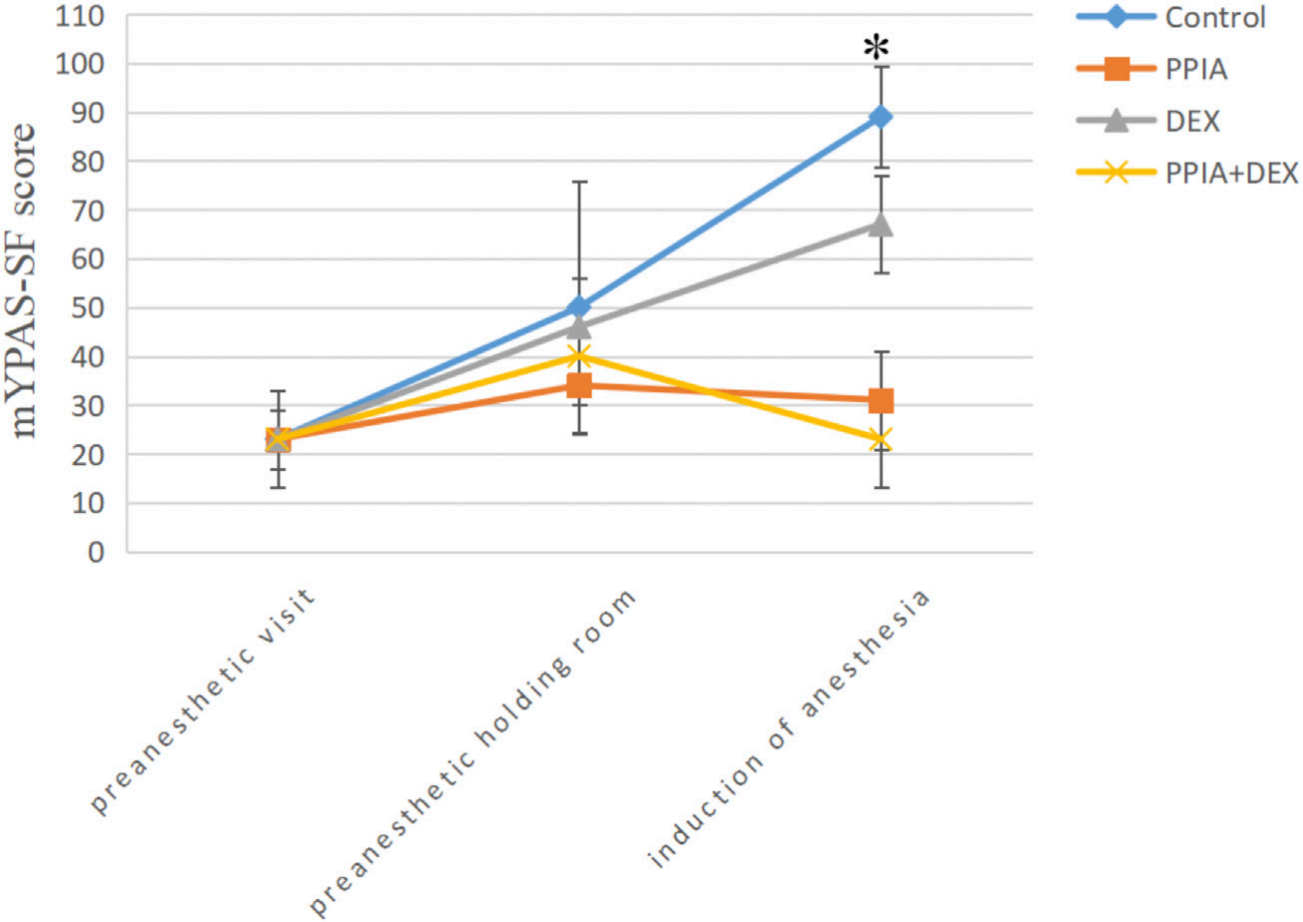
Median mYPAS-SF scores for children over time by group. *p < 0.05 between group analysis. mYPAS-SF (modified Yale Preoperative Anxiety Scale-Short Form); PPIA, parental presence at induction of anesthesia. DEX, intranasal dexmedetomidine.

There was no significant difference in mYPAS-SF scores among the four groups at either T0 or T1 (H = 1.448, *p =* 0.694 or H = 5.838, *p =* 0.140). There was a significant difference in mYPAS-SF scores among the four groups at T2 (H = 53.113, *p* < 0.001); the mYPAS-SF scores of the PPIA, DEX, and PPIA + DEX groups were significantly lower than the mYPAS-SF score of the control group (all *p* < 0.001). The mYPAS-SF score of the PPIA + DEX group was significantly lower than the scores of the PPIA and DEX groups (*p* = 0.043 vs. PPIA group, *p* = 0.009 vs. DEX group) (Table 2). The median mYPAS-SF scores and interquartile range (IQR) in the four groups across three time points are shown in Figure 3. In the control group, there was an upward trend in anxiety levels from T0 to T1 and T2 (Z = 54.069,*p* < 0.001). In the PPIA and DEX groups, the mYPAS-SF scores at T1 and T2 were significantly higher than the mYPAS-SF score of T0 (Z = 17.761, *p* < 0.001 and Z = 41.948, *p* < 0.001). In the PPIA + DEX group, the mYPAS-SF score at T1 was significantly higher than the mYPAS-SF scores at T0 and T2 (Z = 23.649, *p* < 0.001).

**TABLE 2:** Primary outcome measures.

Group	Control (*n* = 30)	PPIA (*n* = 30)	DEX (*n* = 30)	PPIA + DEX (*n* = 30)	^H^	^*^ *p* values
mYPAS-SF(T0)	23,1.5 (23–46)	23,6 (23–46)	23,6 (Kain et al., 1998; McGraw and Kendrick, 1998; Aguilera et al., 2003; Bal et al., 2006; Gazal et al., 2015; Cao et al., 2016; Prabhu and Mehandale, 2017; Li et al., 2018; Miller et al., 2018; Tervonen et al., 2020; Bromfalk et al., 2021; Gil Mayo et al., 2022)	23,6 (23–46)	1.448	0.694
mYPAS-SF(T1)	50,30.25^#^ (23–100)	34,12.5^#^ (23–100)	46,42.5^#^ (23–100)	40,25.75^#^ (23–77)	5.838	0.140
mYPAS-SF(T2)	89,30.5^#,*^ (29–100)	31,45^a#^ (23–100)	67,38.2^ab#^ (23–96)	23,10.25^ab*^(23–77)	53.113	<0.001

Data are Median, IQR (Range); mYPAS-SF, modified Yale Preoperative Anxiety Scale-Short Form; IQR, interquartile range.

*p-value, result by Kruskal–Wallis H test as non-parametric approach. The difference between groups was compared by the Bonferroni test.

mYPAS-SF, scores over time within a group was compared using the Friedman test.

Compared with control group, ^a^ *p* < 0.05.

Compared with group PPIA + DEX, ^b^ *p* < 0.05.

^#^ *p* < 0.05 *vs*. T0 within group.

^*^ *p* < 0.05 *vs*. T1 within group.

### 3.2 Secondary outcome

The compliance of children at anesthesia induction significantly differs among the four groups (H = 44.212, *p* < 0.001). The ICC scores of the PPIA, DEX, and PPIA + DEX groups were significantly lower than the control (all *p* < 0.05). The ICC score of PPIA + DEX was significantly lower than the scores of the PPIA and DEX groups (*p* = 0.043 *vs.* PPIA group, *p* = 0.009 *vs.* DEX group) (Table 2). The children’s PAED was significantly different among the four groups (F = 14.350, *p* < 0.001). The PAED score of the PPIA, DEX, and PPIA + DEX groups were all significantly lower than the PAED score of control (*p* < 0.001 *vs.* PPIA group, *p* < 0.001 *vs* DEX group, *p* < 0.001 *vs.* PPIA + DEX group). There were no statistical differences between the PPIA, DEX, and PPIA + DEX groups. There was no significant difference in the children’s Comfort-B Scale among the four groups at 6 h after the operation (F = 0.535, *p* = 0.659).

In the preanesthetic visit, there was no statistically significant difference in STAI-T scores among the different groups (F = 0.367, *p* = 0.777). However, after induction, the STAI-S scores of the four groups significantly differed (F = 5.380, *p* = 0.002). The STAI-S scores of PPIA, DEX, and PPIA + DEX groups were significantly lower than that of control (*p* < 0.001 *vs* PPIA group, *p* < 0.001 *vs.* DEX group, *p* < 0.001 *vs.* PPIA + DEX group). There was no statistical significant difference between the PPIA, DEX, and PPIA + DEX groups (Table 3). The VAS scores of the four groups were significantly different (F = 16.607, *p* < 0.001). Those of the PPIA, DEX, and PPIA + DEX groups were significantly higher than the score of the control group (*p* < 0.001 *vs.* PPIA group, *p* < 0.001 *vs.* DEX group, *p* < 0.001 *vs.* PPIA + DEX group), and the score of the PPIA + DEX group was significantly higher than those of the PPIA and DEX groups (*p* = 0.013 *vs.* PPIA group, *p* = 0.006 *vs.* DEX group) (Table 3).

**TABLE 3:** Secondary outcome measures.

Group	Control	PPIA	DEX	PPIA + DEX	Effect size	*p values
	(*n* = 30)	(*n* = 30)	(*n* = 30)	(*n* = 30)	H Or F	
ICC	4,3 (0–9)	1,3^ab^ (0–9)	2,3.25^ab^ (0–8)	0,1^a^ (0–6)	H = 44.12	<0.001
PEAD	10.9 ± 4.1	6.7 ± 3.8^a^	6.5 ± 3.8^a^	5.1 ± 2.7^a^	F = 14.350	<0.001
Comfort-B Scale	10.3 ± 3.2	11.1 ± 2.8	10.3 ± 3.1	11.0 ± 3.0	F = 0.535	0.659
STAI-T	45.1 ± 12.8	43.1 ± 10.1	42.1 ± 10.7	43.7 ± 9.9	F = 0.367	0.777
STAI-S	52 ± 11.2	44.2.±11.0^a^	41.9 ± 13.6^a^	41.5 ± 10.0^a^	F = 5.380	0.002
VAS	7,2(5–10)	9,3(5–10)^ab^	9,2(6–10)^ab^	10,1(8–10)^a^	F = 16.607	<0.001

Values are Median, IQR (Range) or mean ± SD (standard deviation).

ICC, Induction Compliance Checklist; PAED, Pediatric Anesthesia Emergence Delirium Scale; COMFORT-B Scale, The COMFORT Behavior Scale; STAI-T, State-Trait Anxiety Inventory–Trait Anxiety; STAI-S, State-Trait Anxiety Inventory–State Anxiety; VAS, Visual Analog Scale; IQR, interquartile range.

^*^ *p*-value, ICC, result by Kruskal–Wallis H test as non-parametric approach. The difference between groups was compared by the Bonferroni test.

Other outcome measures are as a result of one-way analysis of variance (ANOVA). The difference between groups was compared by the LSD test.

Compared with control group, ^a^P<0.001.

Compared with group PPIA + DEX, ^b^P<0.05.

### 3.3 Adverse effects

No anesthetic complications (e.g., laryngospasm) occurred during the inductions. In the PACU, the incidence of vomiting was similar among the four groups (*p* > 0.05).

## 4 Discussion

In this study, we found that PPIA and intranasal dexmedetomidine alleviated the anxiety state induced by anesthesia in the children and parents during emergence delirium in PACU, increasing the children’s compliance as well as parental satisfaction. Compared with PPIA and intranasal dexmedetomidine, PPIA combined with intranasal dexmedetomidine is more effective than either alone.

Contrary to children in other countries, in China the fear of hospital is caused by the traditional cultural environment. Images of a doctor or nurse performing venepuncture are often used to deter naughty children, so the fear of anesthesia and surgery is difficult to eliminate. Although there are many ways to mitigate children’s preoperative anxiety—such as clown doctors, transport in a toy car, tablet games, cartoon videos, and comical information leaflets (Arai et al., 2007; Lee et al., 2012; Kim et al., 2015; Kassai et al., 2016; Marechal et al., 2017; Liu et al., 2018; Lopes-Júnior et al., 2020), some children may still resist the inhalation of stimulating sevoflurane, cry and fight against mask induction, and fall asleep with fear, which may cause delirium at emergence. Intranasal dexmedetomidine in the holding area could make for a smooth separation from parents and transportation to the OR; however, once awakened by the smell of sevoflurane, the children will find themselves in a strange environment and are afraid. PPIA is the most frequently studied non-pharmacologic intervention for reducing children’s preoperative anxiety and is desired by many parents.

Although the effect of PPIA is controversial, many pediatric anesthesiologists believe that parental company is beneficial. It is recommended by the Association of Anaesthetists of Great Britain and Ireland that parents should be invited to accompany their child at the induction of anesthesia. Studies have shown that PPIA can effectively alleviate anxiety during this procedure (Sadeghi et al., 2017). Hatice K. Ozdogan (2017) determined that lower salivary cortisol levels was evidence of reduced anxiety of children in the maternal presence during anesthesia induction, compared with her absence. Varughese et al. (2008) reported that more than half of pediatric patients demonstrated increased compliance with anesthesia induction when their parents were present. During anesthesia induction in the PPIA group, a parent’s presence made children feel safe during anesthesia induction, Most children were able to be induced by sevoflurane inhalation with good compliance, but a few still resisted the anesthesia mask with crying. Dexmedetomidine alone could be used before surgery to produce mild sedation similar to physiological sleep (Baier et al., 2016). Most children could be induced by sevoflurane inhalation with sedation, but some woke from a sedative state when being transferred to the operating bed, or from compression by the anesthesia mask, resulting in anxiety and decreased compliance; Xinlei Lu (2021) reported similar findings. Some studies indicate that dexmedetomidine nasal drops before operation can reduce EC(50) of sevoflurane in laryngeal masks by 21% (Savla et al., 2014). The combination of PPIA and dexmedetomidine was superior to either alone. On the one hand, the pharmacological effect of dexmedetomidine accelerates the induction time of inhalation anesthesia. On the other hand, when the children were induced by sevoflurane inhalation, the parent could calm them even if they woke up.

We found no difference in children’s anxiety scores at T0 or T1 between groups. However, the children’s anxiety score at T0 was lower than that of T1, so staying in a holding room was more stressful than being in the ward. The children’s anxiety score at T2 in the control group was higher than in other groups. This is because, without a parental presence or the preoperative sedative, the children were anxious about the unfamiliar environment and the mask against their face. Giath Gaza et al. (2015) reported that inhalational induction produced more levels of distress than intravenous induction in children, while Aguilera et al. (2003) reported that children were more anxious during intravenous induction than inhalation induction. Bal et al. (2006) showed that both inhalation and intravenous induction of anesthesia led to high levels of anxiety on induction. However, one week post-surgery, intravenous induction showed fewer behaviors linked to psychological trauma. In our hospital, the anesthesiology team of ENT surgery is used to administer inhalation anesthesia because most of the patients are around 2–4 years old and are very afraid of needles. Although there is resistance to induction of inhalation anesthesia, it is painless and easier. When the mask is close to and against the children’s face, they become hypomanic and anxious. Therefore, whether in intravenous or inhalation induction of anesthesia, the mYPAS-SF score at induction in the control group will be high. In the study by Bromfalk et al. (2021), there were lower mYPAS and ICC scores with preoperative sedation, which were lower than the score in intranasal dexmedetomidine in our research. Perhaps 2 μg/kg intranasal dexmedetomidine in their study is more effective than 1 μg/kg intranasal dexmedetomidine in our research, and sufficient preoperative sedative is more useful than any other method. While Kain et al. (1998) concluded that oral sedative has a better effect than PPIA, we found it to have a similar effect on intranasal dexmedetomidine. Perhaps this is because both Drs. Sadeghi and Kain used midazolam as the sedative drug whereas we used dexmedetomidine.

Midazolam is a benzodiazepine that is widely used as a sedative premedicant in patients. The advantages of midazolam include pharmacological sedation, anxiolysis, and anterograde amnesia. Studies have shown that midazolam can improve compliance during induction and decrease the incidence and intensity of emergence delirium (Gil Mayo et al., 2022). However, intranasal midazolam with burning sensation in the nasopharynx and oral midazolam with a bitter taste are strong irritants to children. Some studies have shown that children who received midazolam experienced delayed recovery and more adverse postoperative behavior changes (McGraw and Kendrick, 1998).

Dexmedetomidine is a novel, highly selective, specific α2-adrenergic receptor agonist that produces sedative, analgesic, anxiolytic, and sympathetic inhibition with no significant respiratory depression. The pH value of dexmedetomidine is 4.5–7: less irritating to the nasal mucosa. It has become increasingly popular for premedication in children. Miller et al. (2018) found that the bioavailability of dexmedetomidine by nasal drip in children was reported to be 83.8%, and there was no difference in the bioavailability of dexmedetomidine by nasal drops or atomization, both of which achieved the same sedation depth (Li et al., 2018). Studies have shown that, compared with premedication with oral midazolam, oral dexmedetomidine provides smooth induction and recovery, reduces emergence agitation, and provides better analgesia and sedation (Prabhu and Mehandale, 2017). A meta-analysis found that intranasal dexmedetomidine was a safe and effective sedative for minor pediatric procedures (Tervonen et al., 2020). So, instead of midazolam, we propose dexmedetomidine as more suitable for combination with PPIA in this study.

Postoperative emergence delirium has been reported in 12%–18% of all children (Cao et al., 2016). Although emergence delirium during the recovery period is self-limiting, the agitated child will prolong the observation time in the PACU and reduce its turnover efficiency. We found similar results to Kain that not only dexmedetomidine but PPIA alone or combined use of PPIA and intranasal dexmedetomidine has a beneficial effect on alleviating emergence delirium in children (Arai et al., 2007). Kain et al. (2004) retrospectively studied the correlation between preoperative anxiety levels and emergence delirium during the anesthetic recovery period. The regression analysis of 1613 children showed that their agitated emergence behavior during the anesthetic recovery period increased by 10% with every 10-point increase in the anxiety score. Therefore, PPIA and intranasal dexmedetomidine reduce emergence agitation by alleviating children’s preoperative anxiety. Yuen et al. (2010) found that the median (95% CI) duration of 1.0 μg/kg dexmedetomidine was 85 (55–100) min while the average operating time in our study was around 40 min. When children were transferred to the PACU, the dexmedetomidine was still functioning, thus reducing the level of emergence delirium during anesthetic recovery.

In terms of alleviating parental anxiety, there was no significant difference in the anxiety level of the parents among the four groups before anesthesia induction. After that, PPIA or intranasal dexmedetomidine alone and in combination alleviated parental anxiety levels during anesthesia induction. When the parents participate in the anesthesia induction process and see the children being transported to OR in a calm state, their anxiety was relieved, leading to higher satisfaction with the medical services. Some studies have shown that most parents prefer to be present during anesthesia induction, regardless of age and previous surgical experience (Braude et al., 1990).

Nowadays in China, with the rapid growth in the amount of surgery, anesthesiology departments are facing enormous challenges, such as imbalanced development among the regions, heavy workloads, and the limited anestheology workforce (Zhang et al., 2021). Despite the heavy workloads, we should also pay attention to the mental health of the children and parents during the perioperative period. Both PPIA and preoperative sedation are beneficial for alleviating the anxiety level of children and improving the satisfaction of parents; this helps build a harmonious relationship between patients and doctors, and makes medical services are more humane.

There are a few limitations in our work. The first is that the children had different waiting times before surgery. The first children, who had surgery in the morning, and the children who waited for surgery in the afternoon, may have had different levels of anxiety because children who had surgery in the afternoon experienced a longer fasting time. Older children understood that they were to have surgery, so the longer they waited, the more anxious they became. However, most of the children began to worry and fear when they entered the OR. Therefore, performing pediatric surgery before noon will also reduce the anxiety level of children. The second limitation is that parents were present during anesthesia induction for both the PPIA and the PPIA + DEX groups, which was unavoidable. Therefore, complete double blindness of the observer is impossible. The third limitation is that there was no psychological assessment of the children for a longer period of time or observation of postoperative behavioral changes in the children. The literature suggests that children are nervous and anxious during anesthesia induction, which may lead to adverse psychological effects after surgery. The probability of behavioral change after surgery increases by 12.5% with every 10% increase in the mYPAS score; 67% of children will have behavioral changes after surgery, which will last six months in 20% of children and one year in 7%. Studies have also shown that PPIA and preoperative sedatives have no significant effect on adverse behavioral changes two weeks after surgery. This experiment failed to assess the longer-term indicators, and it is impossible to determine whether there is an advantage of PPIA and intranasal dexmedetomidine alone or in combination after a certain period of time. However, most of the previous studies evaluated pediatric general surgery, in which the children had substantial surgical trauma and extended postoperative pain. The surgery in this experiment was of the tonsils or adenoids. The operation time was short and the trauma was minimal, which allowed the children to recover quickly. The experimental data showed no significant difference in comfort level at 6 h after the operation among different groups. The children achieved complete psychological recovery after seven days. We did not study the psychological and behavioral changes for a longer period after surgery.

In conclusion, both PPIA and intranasal dexmedetomidine may alleviate the preoperative anxiety of children and parents, children’s emergence delirium, and improve children’s induction compliance and parental satisfaction. The combined use of PPIA and intranasal dexmedetomidine is more effective than either PPIA or intranasal dexmedetomidine alone for alleviating preoperative anxiety in children and improving children’s induction compliance and parental satisfaction.

## Data Availability

The original contributions presented in the study are included in the article/supplementary material; further inquiries can be directed to the corresponding author.
